# Battery Cathode with Vertically Aligned Microstructure Fabricated by Directional Ice Templating

**DOI:** 10.1002/smsc.202500198

**Published:** 2025-05-22

**Authors:** Guanting Li, Jin Su, Chun Huang

**Affiliations:** ^1^ Department of Materials Imperial College London London SW7 2AZ UK; ^2^ The Faraday Institution Didcot OX11 0RA UK; ^3^ Research Complex at Harwell Rutherford Appleton Laboratory Didcot OX11 0FA UK

**Keywords:** aqueous solvents, electrode microstructures, fabrications, ion diffusions, manufacturing

## Abstract

Conventional slurry coating (SC) makes battery electrodes with random microstructure containing tortuous pores that restrict lithium ion diffusion and reduce battery capacities at faster discharge rates. Herein, a novel directional ice templating (DIT) is developed to make LiNi_0.8_Mn_0.1_Co_0.1_O_2_ (NMC811) cathodes that double the electrode mass loading and contain vertically aligned lamellae of electrode materials and pore channels to provide fast dual electron and ion transport. DIT uses in situ evolved ice structures to form the anisotropic microstructure. The effects on the chemical composition, bonding, and morphology of the NMC811 particles are studied using a range of surface‐sensitive techniques including time‐of‐flight secondary ion mass spectrometry, transmission electron microscopy, and X‐ray photoelectron spectroscopy to guide the development of potentially more sustainable aqueous processing and eliminate the toxic, combustible organic solvent N‐methyl‐2‐pyrrolidone in conventional electrode processing. The DIT cathode breaks the trade‐off between high energy densities and fast discharging, exhibiting higher areal capacities (12 mAh cm^−2^) than the SC electrode (7.0 mAh cm^−2^) at a discharge current density of 1.4 mA cm^−2^, and maintains higher capacities at 9.8 mAh cm^−2^ and 186 mAh g^−1^ than 2.1 mAh cm^−2^ and 64 mAh g^−1^ for SC when the current is increased to 5.7 mA cm^−2^.

## Graphical abstract

Directional ice templating (DIT) makes NMC811 cathodes containing vertically aligned lamellae of electrode materials and pore channels for fast electron and ion transport. The porosity of DIT electrodes was compatible to conventional slurry coated electrodes. The aqueous processing led to high cathode performance and eliminated toxic organic solvent.

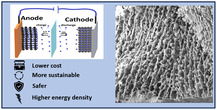

## 1 Introduction

Lithium‐ion batteries (LIBs) are critical in transitions to clean energy due to their versatile and efficient energy storage capabilities.^[^ ^1^ ^]^ LIBs play a vital role in the electric automotive industry,^[^ ^2^ ^]^ consumer electronics, and grid energy storage^[^ ^3^ ^]^ from intermittent renewable sources.^[^ ^4^ ^]^ Recent LIB research trends include energy density improvement, fast charging, high longevity, and stable cycle lives. A LIB consists of several key components, including a cathode, an anode, a separator, an electrolyte, and current collectors.^[^ ^5^ ^]^ Lithium ions diffuse between the cathode and anode during discharging.^[^ ^6^ ^]^ The cathode releases lithium ions that diffuse to the anode during charging, and the anode releases lithium ions, which are intercalated back to the cathode material during discharging.^[^ ^7^ ^]^ LiNi_0.8_Mn_0.1_Co_0.1_O_2_ (NMC811) has gained significant attention as a cathode active material for LIBs because of its high theoretical capacities and a higher voltage platform of ≈3.6 V than that of LiFePO_4_ (3.2 V).^[^ ^8^, ^9^ ^]^

Electrode processing methods can significantly impact electrode microstructure and LIB performance.^[^ ^10^, ^11^ ^]^ In the conventional cathode manufacturing method of slurry coating (SC), the slurry is made by mixing NMC811 particles with electrical conductivity enhancer (usually carbon nanoparticles) and binder usually in a toxic, combustible, organic solvent N‐methyl‐2‐pyrrolidone (NMP),^[^ ^12^, ^13^ ^]^ then a doctor blade or a similar equipment is used to coat the prepared slurry on an aluminum foil followed by drying and calendar steps.^[^ ^14^, ^15^ ^]^ This method helps hold the particles together and adheres them to the current collector.^[^ ^16^ ^]^ Conventional SC electrodes are 100–200 μm in thickness; higher electrode thicknesses and mass loadings can increase the proportion of electrode active materials and increase battery energy densities at the cell‐stack level within a confined volume.^[^ ^17^, ^18^ ^]^ However, the capillary force generated in the drying process by heating causes electrodes to crack and prevents SC from making thicker (≥340 μm) electrodes. Furthermore, the electrode microstructure made by the SC method is not controlled well,^[^ ^19^ ^]^ the congested particle arrangement with tortuous pores prevents efficient electrolyte penetration and ion diffusion, and this problem is exacerbated at higher thicknesses.^[^ ^20^ ^]^ Poor lithium‐ion diffusion limits the contact between the active electrode material particles and the electrolyte,^[^ ^21^, ^22^ ^]^ hindering the ability of lithium ions moving into and out of the electrode material particles and impairing the rate capability of the cell, especially at faster discharge rates.^[^ ^23^, ^24^ ^]^ Some studies focus on investigating component‐interface interactions and the mechanical behavior of electrodes, such as studying mechanical relaxation behavior and bending capability,^[^ ^25^ ^]^ highlighting the importance of the active material microenvironment (ME@AM).^[^ ^26^, ^27^ ^]^ Therefore, optimizing electrode microstructure is imperative for enhancing the overall performance and efficiency of LIBs.^[^ ^28^, ^29^, ^30^ ^]^ Many studies aim to develop new active materials and apply additives to battery components,^[^ ^31^ ^]^ but less attention is paid to the processing to control the hierarchical nano‐ and microstructure.^[^ ^32^, ^33^ ^]^

In this work, we show a novel, rapid bottom‐up approach of directional ice templating (DIT) to make NMC811 cathodes with high thickness (660–1100 μm) and control the anisotropic cathode microstructure containing alternating vertically aligned lamellae of electrode materials (10–15 μm in width) and pore arrays (6–12 μm in diameter) through the electrode thickness to provide fast dual electron and lithium‐ion transport pathways and thus to improve the battery energy density and C ‐rate capability at the cell‐stack level simultaneously.^[^ ^34^ ^]^ Compared with other methods such as 3D printing, laser structuring, and so on, the advantages of the DIT method include (i) scalability (we can use the scalable slurry coater and simply apply a directional freezing temperature gradient from the bottom during rapid formation of in situ ice template), (ii) versatility (this method can be applied to a wide range of cathode and anode materials), and (iii) structure controllability (the pore channel diameter and porosity of the structure can be controlled by the solid concentration of electrode slurry). We have developed the DIT method for other materials of LiCoO_2_^[^ ^33^ ^]^ and LiFePO_4_,^[^ ^35^ ^]^ and the directional freezing and polymerization method for NMC811 in polymer‐based solid batteries.^[^ ^36^ ^]^ Different active material sizes and surface characteristics resulted in slightly different pore channel diameters and tortuosity, but the vertically aligned microstructure was maintained. Herein, we develop the aqueous DIT method for the lithium‐rich NMC811 cathode materials for the first time. There are two major challenges associated with using DIT for making NMC811 cathodes: one is the aqueous processing of NMC811, and the other is reducing porosity of the DIT method to increase battery energy density. This paper addresses both of the key challenges.

First, the solvent of the slurry is changed from NMP, which is usually used in the SC of cathodes, to water in DIT, which can improve processing sustainability.^[^ ^37^ ^]^ We systematically studied the effects of the aqueous processing on the NMC811 properties. Any residual water remaining in the electrodes after electrode drying or cycling at voltages over the upper voltage limit (e.g., 4.6 V^[^ ^8^, ^38^ ^]^) may cause side reactions of water with LiPF_6_ in the electrolyte that will harm electrolyte stability and ultimately battery capacities.^[^ ^39^, ^40^ ^]^ To address the first challenge, all water was rigorously removed from the DIT cathode directly from ice to vapor during ice sublimation before battery assembly in our experiments to prevent any residual water. Although water in contact with the Ni_x_Mn_y_Co_z_O_2_ (NMC) particles during processing may cause side reactions with NMC811 such as lithium leaching after prolonged exposure to water,^[^ ^41^ ^]^ this is usually due to the long term exposure rather than the rapid process of DIT, e.g., a research showed long term (>28 days) exposure to the ambient environment (CO_2_, H_2_O, and O_2_) may affect the performance of high‐nickel NMC (Ni wt% among all transition metals >80%) materials due to the formation of coating consisting of carbonate, hydroxide species, and enrichment of lithium ions on the particle surface.^[^ ^42^ ^]^ In contrast, aqueous SC within a controlled processing environment has been demonstrated on NMC811 materials, showing water does not affect the electrochemical properties of NMC811.^[^ ^43^ ^]^ We developed the DIT process to involve very rapid nucleation and growth of ice and used a high solid concentration slurry to minimize the contact between the NMC811 particles and water.^[^ ^42^, ^44^ ^]^ We used a variety of surface‐sensitive techniques to study the chemical composition, bonding, and morphology of the top 35 nm of the particle surface under different conditions. We showed that the properties of the NMC particles exposed to water within the duration and controlled environment of DIT were compatible with those of the pristine NMC particles in ambient conditions.

Second, DIT usually makes samples with a high porosity (50–60%),^[^ ^45^ ^]^ which may reduce energy densities at the practical cell‐stack level for real battery applications. We studied changing electrode slurry concentrations as an effective method to reduce porosity to 35% to be compatible with the conventional SC electrodes while creating the aligned microstructure for the DIT electrodes. We systematically compared the chemical, nano‐ and microstructural, and electrochemical properties of the NMC811 cathodes made by DIT and conventional SC. Notable differences emerged across various charge rates, at a discharge rate of 0.1 C (the discharge current density was 1.4 and 0.76 mA cm^−2^ for the DIT and SC electrodes, respectively, due to the almost doubling of electrode thickness and mass loading of the DIT compared with SC), the DIT cathode exhibited an areal capacity of 12 mAh cm^−2^, and a gravimetric capacity of 201 mAh g^−1^, compared to an areal capacity of 7.0 mAh cm^−2^ and a gravimetric capacity of 200 mAh g^−1^ for the cathode made by SC. The advantage was already obvious when the discharge rate was increased to 0.4 C (the discharge current density was 5.7 and 2.9 mA cm^−2^ for the DIT and SC electrodes, respectively), the DIT cathode exhibited a higher areal capacity of 9.8 mAh cm^−2^ and gravimetric capacity of 186 mAh g^−1^, compared to 2.1 mAh cm^−2^ and 64 mAh g^−1^ for the SC cathode. At higher C rates of 0.5, 1, and 2 C, the DIT cathode also maintained higher areal and gravimetric capacities than the SC electrodes. In this work, we highlight the following key contributions: (i) developing aqueous processing for the NMC811 material within the DIT processing window to improve processing sustainability; and (ii) overcoming low porosity that is usually associated with the DIT processing method and achieving higher electrode densities compared to the previous electrodes, e.g.^[^ ^35^ ^]^ and increasing volumetric capacity and energy density.

## 2 Results and Discussion

### 2.1 Fabrication of Cathodes by DIT

**Figure 1:**
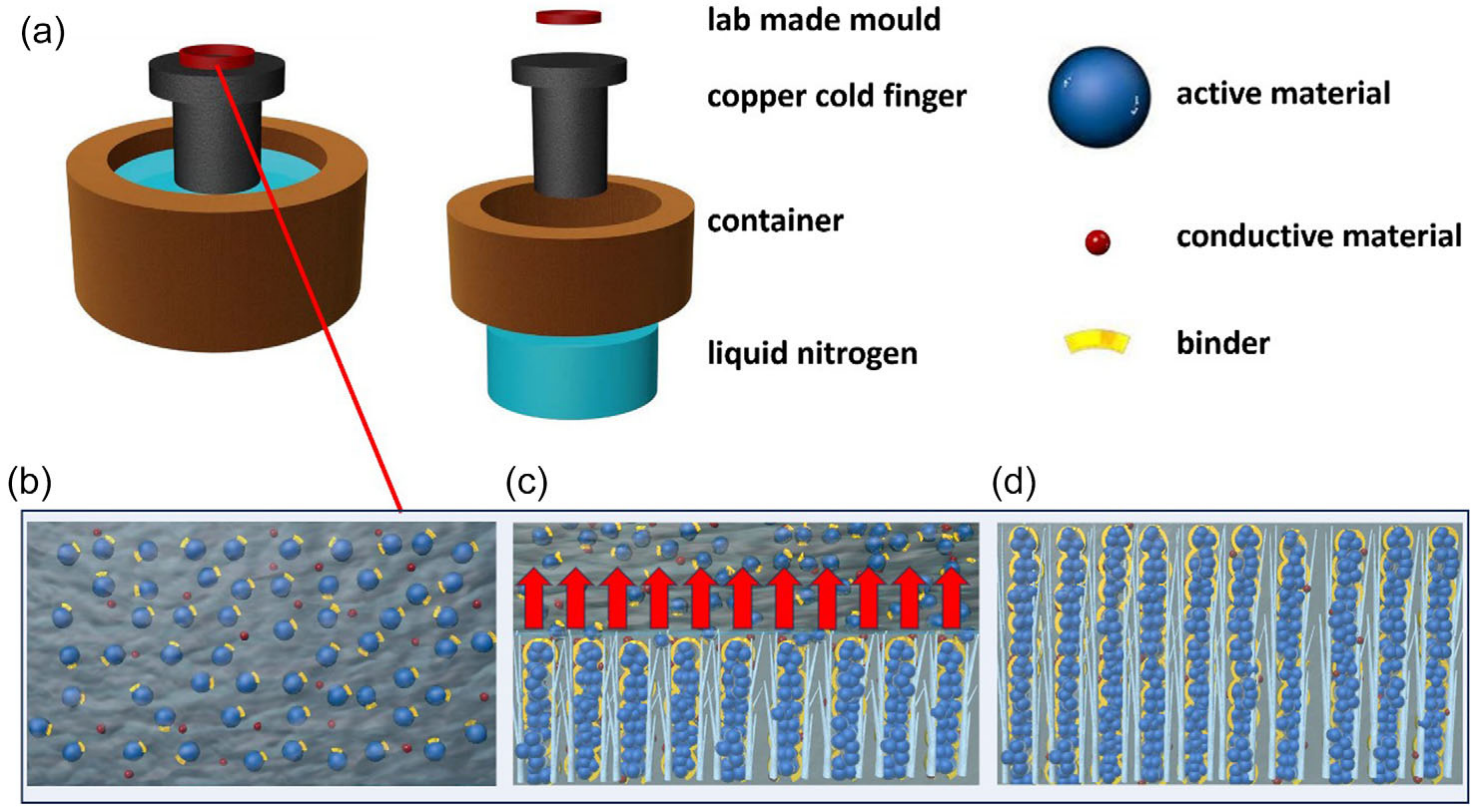
a) Schematics of the DIT apparatus, and the DIT processing steps showing b) initial electrode slurry state, c) slurry during directional freezing, and d) fully frozen slurry.

The slurry composition consisted of NMC811 (90 wt%) as the active material, complemented by carbon black nanoparticles (5 wt%) serving as the conductive element,^[^ ^46^ ^]^ and mixed binders of styrene‐butadiene rubber (SBR, 3 wt%) and carboxymethyl cellulose (CMC, 2 wt%).^[^ ^47^ ^]^ The weight ratio between the solid mixture and deionized water was 30:70 and 40:60 for the dilute and concentrated slurry, respectively. We denote the electrodes as DIT‐D (electrode made by the dilute slurry) and DIT‐C (electrode made by the concentrated slurry), respectively. We investigated the effects of solid concentration in electrode slurry on controlling pore channel diameter and porosity. The lab‐made apparatus of the DIT method integrates three parts, including a lab‐designed round acrylonitrile–butadiene–styrene (ABS) mold for the electrode slurry, a copper cold finger, and a container of liquid nitrogen.^[^ ^48^ ^]^ The ABS mold was placed on top of the copper cold finger platform, which was placed in the container (**Figure** **1**a). The electrode active material particles and electrical conductivity enhancer C nanoparticles with polymeric binder were first homogeneously mixed in the electrode slurry (Figure 1b). During the DIT process, the electrode slurry experienced rapid vertical directional freezing from underneath. Ice crystals started to nucleate at the supercooling interface on the copper cold finger. Ice columns started to grow vertically from the bottom along the freezing temperature gradient. As these dendrites grew along the vertical freezing direction, the ice dendrites exerted pressure on the NMC811 and carbon particles, displacing them into the columns in between the ice dendrites and in situ shaping them into the desired microstructure (Figure 1c). As heat was efficiently taken away from the electrode slurry through conduction, ice columns continued to grow vertically through the entire electrode thickness (Figure 1d). After the electrode reached full solidity, the ice columns were directly sublimed from solid to vapor through freeze‐drying to preserve the formed microstructure. The critical step of ice sublimation prevented the ice dendrites from transitioning back into a liquid phase before ice removal, and hence, prevented the effect of shrinkage that usually takes place in conventional SC electrodes due to the capillary force during conventional evaporation of solvent from liquid to vapor through heating.^[^ ^49^ ^]^ Thus, we were able to make ultra‐thick (660–1100 μm) DIT cathodes without cracking to increase mass loading of the electrodes while maintaining the designed internal microstructure.

The reference electrode group was made by the conventional SC method.^[^ ^7^ ^]^ The average thickness of the SC electrode was 340 μm with an average mass loading of 36 mg cm^−2^. The reason for using this thickness was that this was the highest thickness (for fair comparison in ion diffusion kinetics with the ultra‐thick DIT electrodes) made by SC without cracking due to the capillary forces during heat drying. The average thicknesses of DIT‐C and DIT‐D electrodes were 660 and 1070 μm, respectively, with the same average mass loading of 70 mg cm^−2^. Note that we deliberately made the DIT electrodes thicker to demonstrate that even at this high thickness, ion transport kinetics through the electrode thickness were still higher than the thinner conventional SC electrodes. In real battery applications, the DIT electrode thickness can be modified according to the requirements of specific applications.

### 2.2 Cathode Active Material Particle Surface Properties

**Figure 2:**
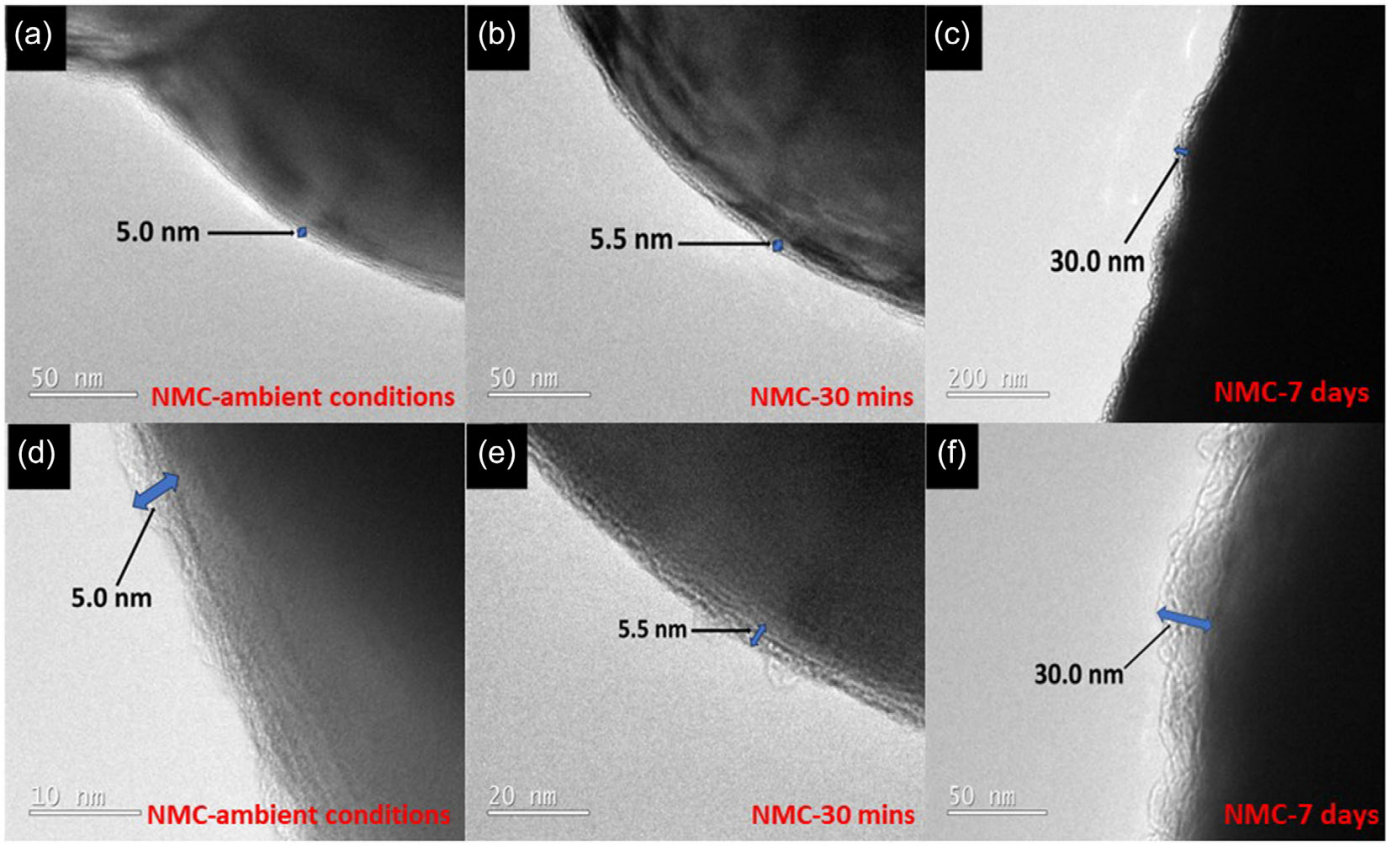
TEM images of the NMC811 particle surface and their corresponding magnified images for a,d) pristine particle in ambient room conditions without deliberate water exposure; b,e) particle immersed in water for 30 min; c,f) particle immersed in water for 7 days.

To investigate the influence of water exposure on NMC811, we investigated three types of electrode for comparison, (i) NMC‐ambient conditions (NMC811 without deliberate water exposure), (ii) NMC‐30 min (NMC811 particles deliberately immersed in water for 30 min to simulate conditions in the DIT method where the NMC811 particles are in contact with water for 25–30 min), (iii) NMC‐7 days (NMC811 particles immersed in water for prolonged exposure of seven days). All the NMC particles then underwent freeze‐drying before being investigated. Transmission electron microscopy (TEM) was employed to investigate the NMC811 particle surface morphology and to quantify the thickness of the coating (if any) on the particle surface from reacting with water. The TEM images in **Figure** **2**a,d show the pristine NMC811 particle in ambient conditions that has not been deliberately exposed to water (NMC‐ambient conditions); the particle displayed a coating thickness of ≈5.0 nm, likely due to the inevitable exposure to CO_2_, moisture, and O_2_ in the ambient environment. The particle immersed in water for 30 min (NMC‐30 min) exhibited a similar coating thickness of ≈5.5 nm (Figure 2b,e), indicating minimal additional reaction compared to the pristine particle. In contrast, the sample immersed in water for 7 days (NMC‐7 days) showed a substantially thicker coating of ≈30.0 nm (Figure 2c,f), suggesting significant surface reactions over the prolonged exposure.

**Figure 3:**
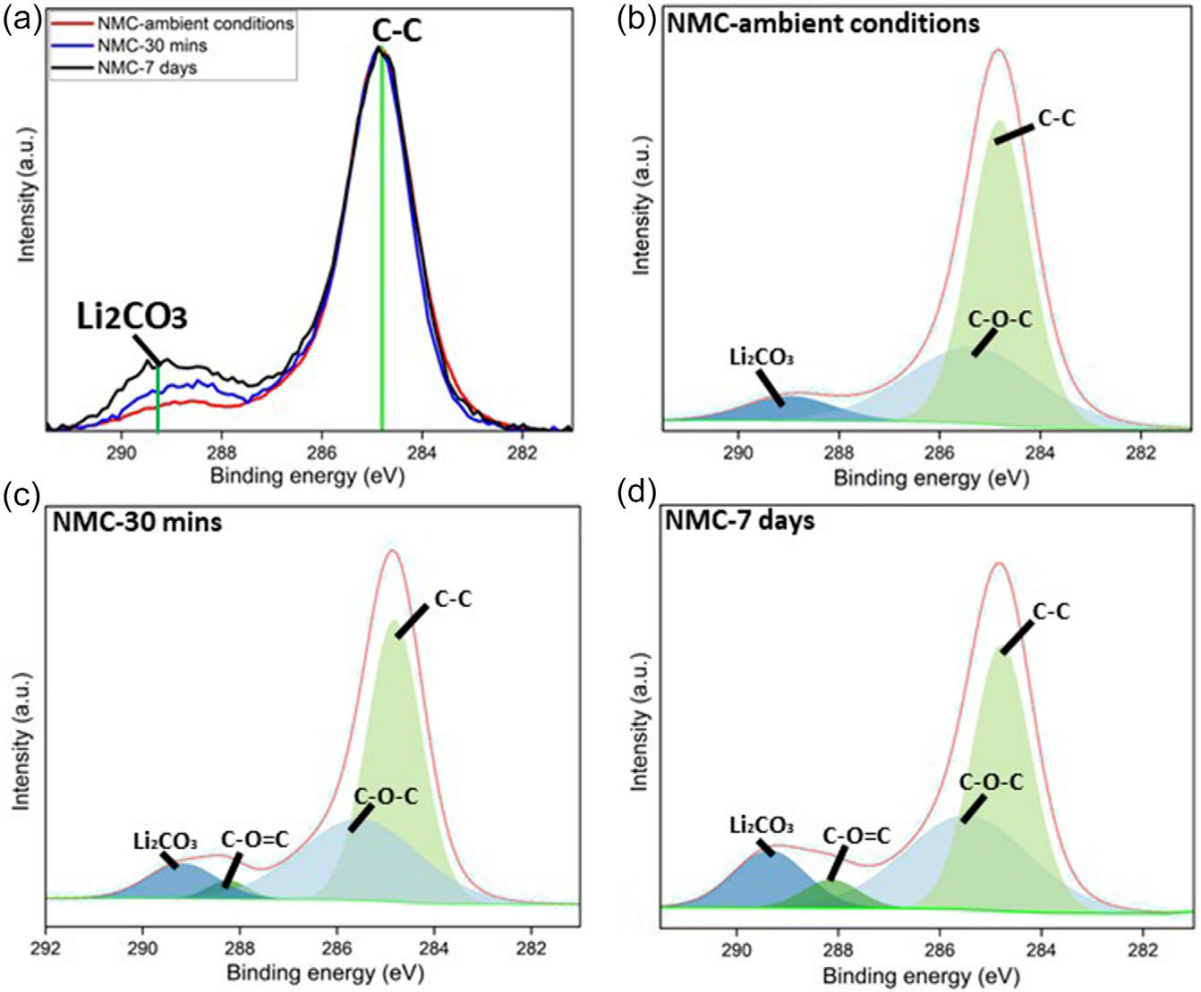
XPS C1s spectra of a) three types of NMC811 particles superimposed together showing the content of Li_2_CO_3_; and the fitted spectra of b) pristine particle in ambient room conditions without deliberate water exposure (NMC‐ambient conditions), c) particle immersed in water for 30 min (NMC‐30 min), d) particle immersed in water for 7 days (NMC‐7 days).

**Figure** **3**a shows the X‐ray photoelectron spectrometer (XPS) C1s spectra for the three types of particles superimposed together, showing the highest intensity of Li_2_CO_3_ was found in NMC‐7 days. The intensity ratio between the Li_2_CO_3_ and C—C peaks was 12.0, 16.0, and 26.6% for NMC‐ambient conditions, NMC‐30 min, and NMC‐7 days, respectively. Figure 3b–d shows the fitted XPS C1s spectra for the three types of particles individually, with the highest intensity of the C—O=C peak for the NMC‐7 days sample.

**Figure 4:**
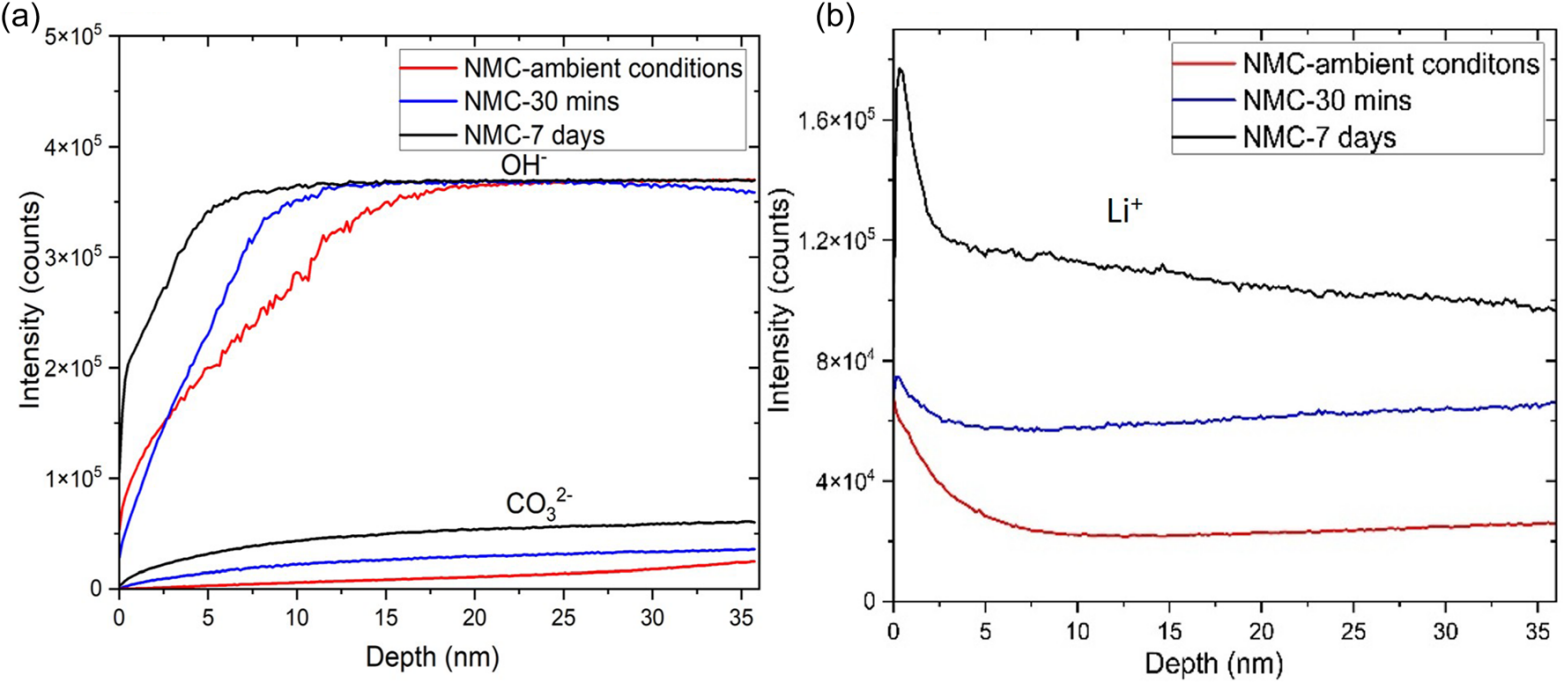
ToF‐SIMS depth profile of a) negatively charged species and b) positively charged species, showing the formation of Li_2_CO_3_ and LiOH from the surface of NMC811 particles to the inside particles under three conditions: NMC‐ambient conditions, NMC‐30 min, and NMC‐7 days.

To provide quantitative analysis of the distributions of lithium ion enrichment and the carbonate and hydroxide species, time‐of‐flight secondary ion mass spectrometry (ToF‐SIMS) depth profiling was conducted on the three types of particles. The samples were sputtered using an ion beam, allowing for depth profiling of the top 35 nm on the particle surface. The ToF‐SIMS depth profiles show the negatively charged CO_3_^2−^ and OH^−^ species content and indicate that NMC‐7 days had the highest intensities of CO_3_^2−^ and OH^−^ at each depth, and NMC‐30 min and NMC‐ambient conditions exhibited much lower intensities (**Figure** **4**a), consistent with the thicknesses of the coatings observed in TEM in Figure 2. Figure 4b presents ToF‐SIMS depth profiles of the positively charged Li^+^ species from the surface to the inside particle of the three types of samples. NMC‐7 days exhibited a significantly higher Li^+^ intensity compared to the other two samples. Corroborating with Figure 4a, the results show a higher presence of Li_2_CO_3_ and LiOH on the particle surface of NMC‐7 days, suggesting that more Li^+^ was leached from NMC‐7 days. The consistency among the TEM, XPS, and ToF‐SIMS results suggests that the coating formed during 30 min of water exposure was comparable to the naturally occurring coating formed under ambient conditions. These results indicate that exposure of NMC811 to water for less than 30 min during aqueous slurry mixing resulted in minimal side reactions compared with the naturally occurring coating for the pristine particles. However, longer exposure time may lead to substantial surface reactions and coating formation.

**Figure 5:**
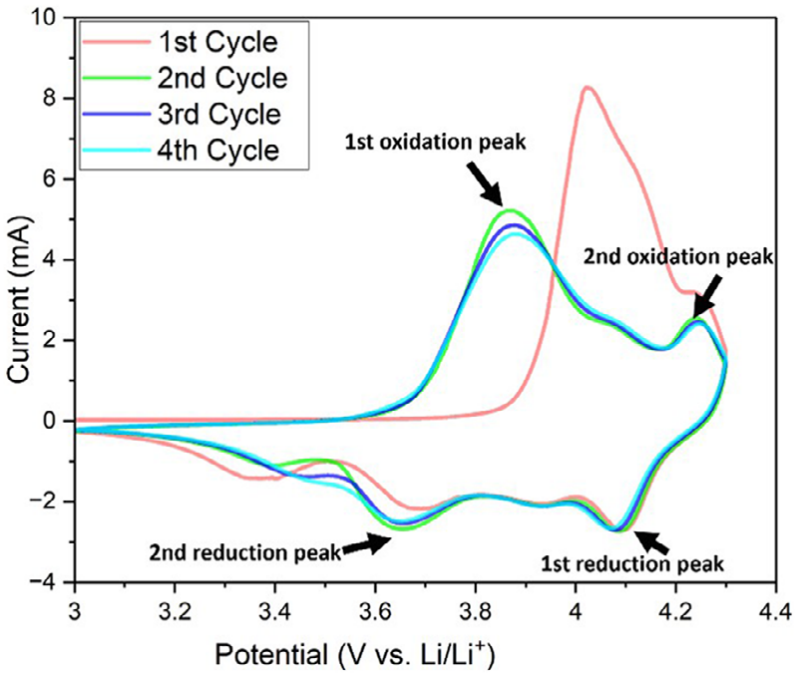
CV curves of DIT‐C electrode at a scan rate of 0.1 mV s.^−1^.

A cyclic voltammetry (CV) test was conducted on electrodes prepared using the DIT method, and the resulting CV curve is shown in **Figure** **5**. In the pristine NMC811 electrode fabricated by the conventional SC method, the stepwise redox behavior of Ni and Co resulted in four distinct peaks—two oxidation and two reduction peaks, each pair corresponding to a specific redox reaction at given potentials. During charging, the first oxidation peak appeared within the 3.7–4.0 V range, corresponding to the removal of Li^+^ from the NMC811 structure, while the second oxidation peak occurred between 4.2–4.5 V due to further Ni oxidation.^[^ ^50^, ^51^, ^52^ ^]^ During discharge, the first reduction peak emerged between 4.1–3.9 V, indicating lithium reinsertion, followed by a second reduction peak in the 3.7–3.6 V range, representing additional lithium insertion. After the first cycle, the formation of a cathode–electrolyte interphase stabilized the system, leading to more consistent and overlapping CV curves in subsequent cycles. As shown in the Figure 5, the CV curves of the NMC811 electrode fabricated by the DIT method at 0.1 mV s^−1^ over the first four cycles exhibited a behavior highly consistent with conventional NMC811. From the second to the fourth cycle, the CV curves overlapped more closely, demonstrating stable electrochemical behavior. The oxidation peaks for these cycles appeared at 3.87 and 3.88 V (first oxidation) and 4.24 and 4.25 V (second oxidation), while the reduction peaks were observed at 4.08 and 4.07 V (first reduction) and 3.65 and 3.62 V (second reduction). This strong agreement with the conventional NMC811 characteristics using NMP solvent further confirms that the aqueous DIT method did not alter the redox reaction mechanism.

### 2.3 Electrode Structures

**Figure 6:**
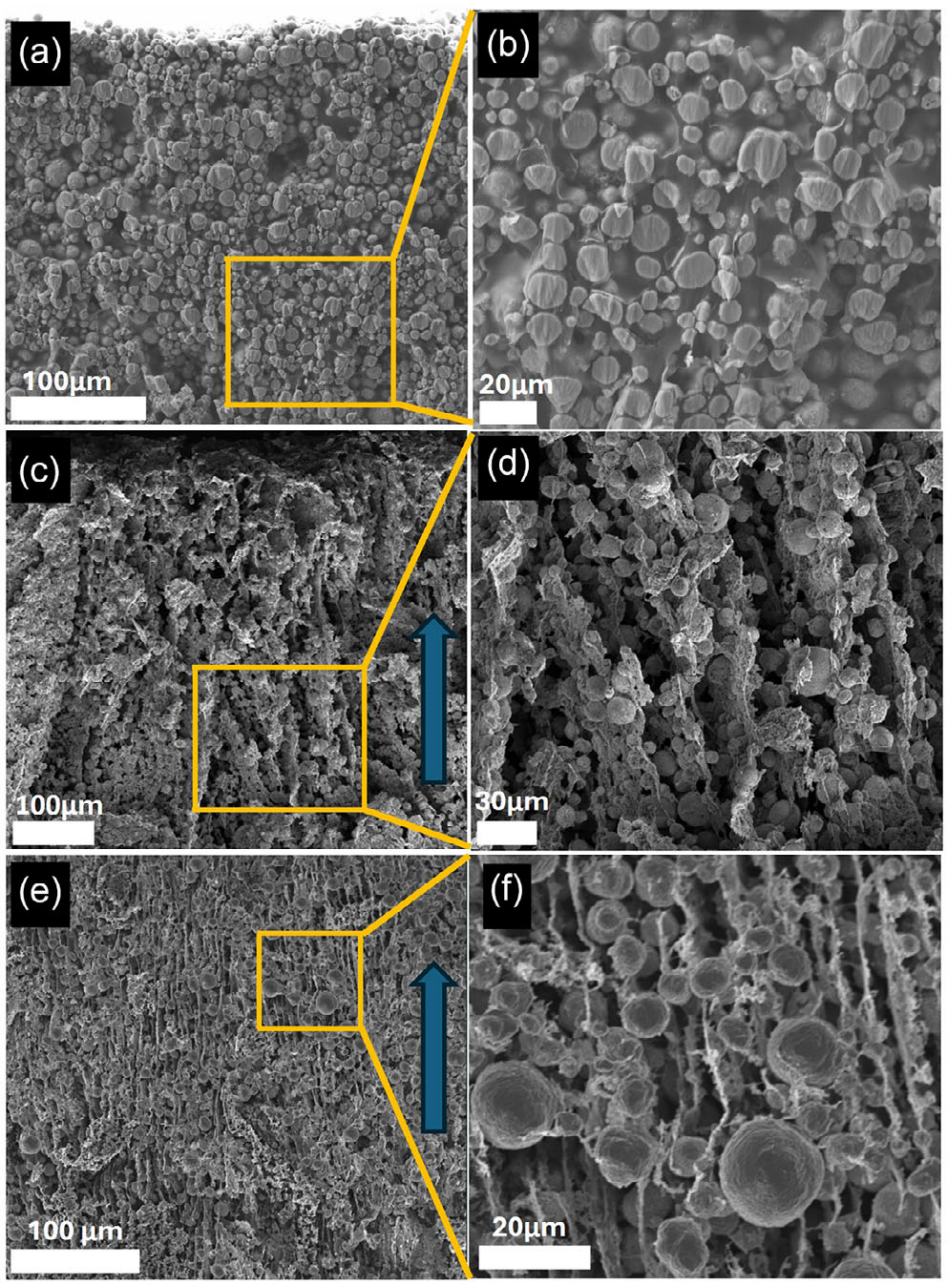
Cross‐sectional SEM images of a,b) electrode made by the SC method; c,d) electrode made by the DIT method using a dilute slurry (solid:liquid wt% = 30:70, DIT‐D); and e,f) electrode made by the DIT method using a concentrated slurry (solid:liquid wt% = 40:60, DIT‐C). The blue arrows in (c) and (e) indicate the freezing direction during DIT.

**Figure** **6** shows the cross‐sectional scanning electron microscopy (SEM) images of the SC electrode, DIT‐D and DIT‐C with increasing magnifications, and the blue arrows in the figures show the freezing direction during the DIT process. The cross sections were polished by an argon ion beam to reveal the interior microstructure of the electrodes. The average secondary particle size of NMC811 was 12 ± 5 μm, while the carbon black particles were around 8–100 nm. Figure 6a,b shows non‐directional porosity with highly tortuous pores for the SC electrode. Figure 6c–f shows well‐ordered pore channels extending through the thickness direction for the DIT‐D and DIT‐C electrodes. These pore channels were formed along the freezing direction during the ice columns’ growth. The vertical structure framework enables shorter diffusion pathways along the kinetically favorable through‐electrode‐thickness direction for ion diffusion during discharging. Individual NMC811 particles were observed in DIT‐C in Figure 6e,f, the width of the lamellae of the electrode materials was 10–15 μm, facilitating efficient insertion and extraction of lithium ions as active sites for electrochemical processes. The SC electrode exhibited a large pore size distribution of 2–30 μm (Figure 6a,b), whereas the DIT‐D (Figure 6c,d) and DIT‐C (Figure 6e,f) electrodes exhibited more uniform pore channel diameters of 4–10 and 2–6 μm, respectively. The overall porosity was 35%, 57%, and 35%, ±5%, for the SC, DIT‐D, and DIT‐C electrodes, respectively, demonstrating that increasing the solid content in the electrode slurry for DIT effectively increased electrode density to be compatible to that of SC electrodes, and modifying the electrode slurry solid concentration can effectively control the pore‐channel size and porosity. The DIT electrodes were also able to be made much thicker (660–1100 μm) than the SC electrode (340 μm), and no obvious cracks were observed in the DIT electrodes, showing good mechanical properties of the DIT electrodes. Additionally, Figure S1, Supporting Information in the Supplementary Information (SI) shows a top view SEM image of DIT‐C (freeze direction was towards out of the page) after ion milling to reveal the internal microstructure, a cellular structure emerged wherein all active material particles were interconnected in the conductive network of the electrode. We have also performed calendering tests on the DIT electrodes, showing that the electrode thickness of porosity was reduced by moderate calendering, the vertically ordered porous structures bended but largely preserved the ordered porous microstructure.

### 2.4 Electrochemical Performance

**Figure 7:**
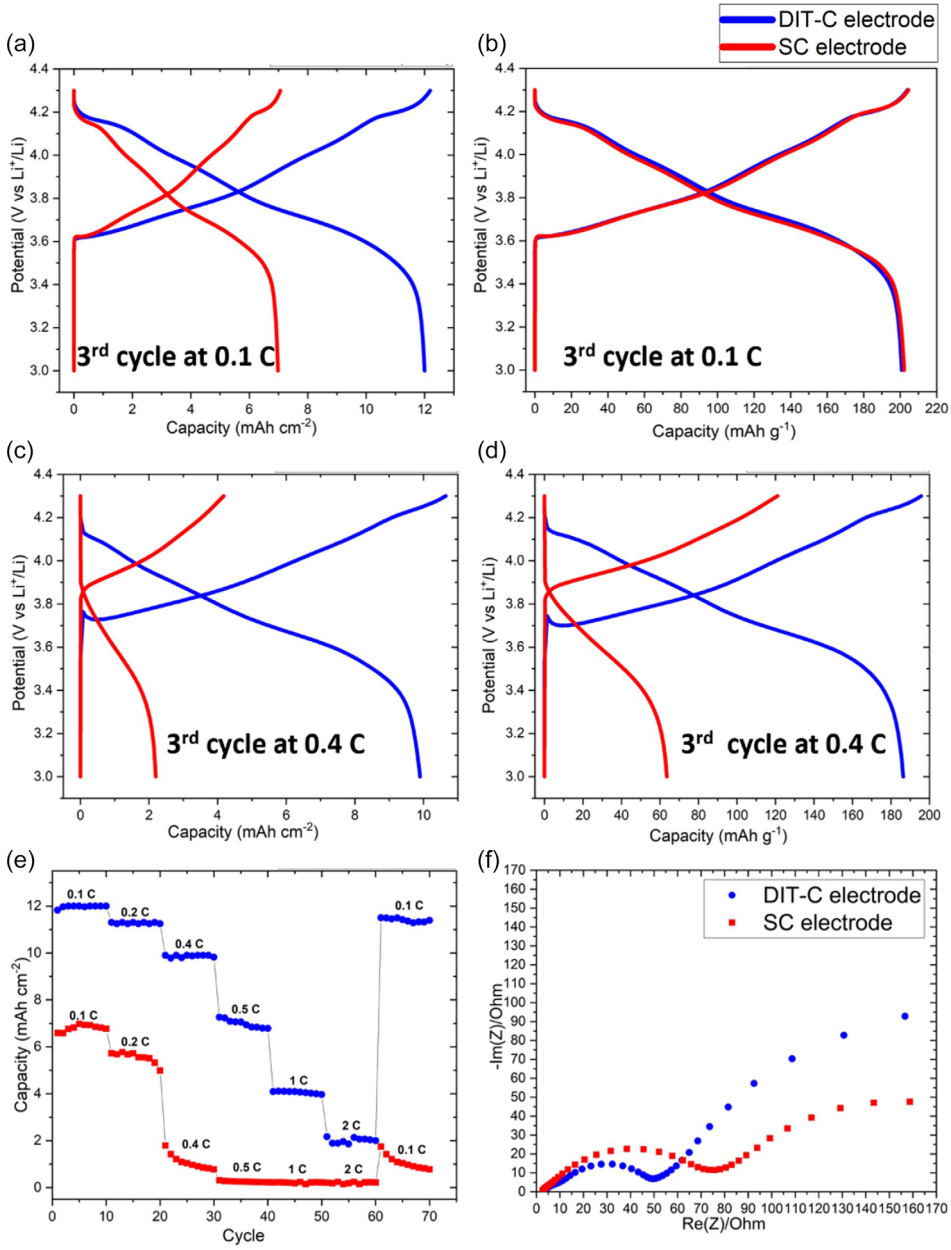
Galvanostatic charge and discharge profiles showing a) areal capacities at 0.1 C; b) gravimetric capacities at 0.1 C; c) areal capacities at 0.4 C; d) gravimetric capacities at 0.4 C. e) reversible areal capacity of electrodes at different C rates. f) Fitted Nyquist plots for comparison between DIT‐C and SC cathodes.

**Figure** **7**a,b shows the galvanostatic discharge profiles for the DIT‐C and SC cathodes, showing both gravimetric and areal capacities at discharge rates of 0.1 and 0.4 C. The mass loading of the DIT‐C cathode is almost double of the thick SC cathode we made for comparison purposes and the mass loading of the DIT‐C cathode is almost four times of the average of the SC cathodes in the literature,^[^ ^53^ ^]^ hence, the magnitudes of the current applied (parameter for practical applications) to the three types of cathode at the same C rate were different. At 0.1 C, the discharge current density was 1.4 and 0.76 mA cm^−2^, and the gravimetric capacities were 201 and 200 mAh g^−1^ for the DIT‐C and our SC cathodes, respectively. The gravimetric capacity of DIT‐C was also comparable to the other thinner NMC811 cathodes made by SC (17 mg cm^−2^) in the literature,^[^ ^53^ ^]^ although the current applied to the DIT electrodes (70 mg cm^−2^) and our SC electrodes (36 mg cm^−2^) was significantly higher than the conventional SC electrodes, demonstrating that the DIT‐C cathode fully utilized its active materials in the aligned microstructure despite the higher thickness. In contrast, due to the higher mass loading of the DIT‐C cathode, DIT‐C significantly increased areal capacity (12 mAh cm^−2^) compared with 7.0 mAh cm^−2^ for the SC electrode in this study and ≈3.25 mAh cm^−2^ for the other SC cathodes in the literature.^[^ ^54^ ^]^ The Coulombic efficiency was 98.5% and 97% for the DIT‐C and SC cathodes, respectively. Figure S2, Supporting Information shows that the DIT‐D cathode exhibited an areal capacity of 9.3 mAh cm^−2^ since it still had higher mass loading than SC, and the vertical pore arrays improved ion diffusion. At 0.4 C, the discharge current densities were 5.7 and 2.9 mA cm^−2^, and the gravimetric capacities were 186 and 64 mAh g^−1^ for the DIT‐C and SC cathodes, respectively. The lower gravimetric capacity of the SC cathode here is because the SC cathode thickness in this study (340 μm) was still 2–3 times higher than that of conventional SC cathodes,^[^ ^55^ ^]^ and the tortuous porosity heavily impeded ion diffusion. In contrast, the DIT‐C cathode still maintained a higher gravimetric capacity and 4 times higher areal capacity (9.8 mAh cm^−2^) than the SC cathode (2.1 mAh cm^−2^). Figure S3, Supporting Information shows the DIT‐D electrode exhibited a medium areal capacity of 8.0 mAh cm^−^^2^ at 0.4 C.

Figure 7e shows the comparison of reversible areal capacities for the DIT‐C and SC cathodes from 0.1 to 2 C (the corresponding reversible gravimetric capacities comparison is shown in Figure S4, Supporting Information. Due to the significantly higher mass loading of the DIT‐C electrode than the SC electrode,^[^ ^56^ ^]^ the current densities applied to DIT‐C (mass loading 70 mg cm^−2^) at 0.1, 0.2, 0.4, 0.5, 1, and 2 C were approximately equivalent to the current densities applied to our SC electrode (mass loading 36 mg cm^−2^) at 0.2, 0.4, 0.8, 1, 2, and 4 C, and the current densities applied to conventional SC electrodes in the literature (average mass loading 17 mg cm^−2^^[^ ^57^ ^]^ at 0.4, 0.8, 1.6, 2, 4, and 8 C). At the same current density of 1.4 mA cm^−2^ for a fair comparison, the DIT‐C electrode exhibited 200 mAh g^−1^ whereas the SC electrode exhibited 182 mAh g^−1^; at the same current density of 1.4 mA cm^−2^, the DIT‐C electrode exhibited 156 mAh g^−1^ whereas the SC electrode exhibited 140 mAh g^−1^;^[^ ^58^ ^]^ and the difference became larger and larger as the current density increased, showing the advantage of the DIT electrode. The areal capacity of DIT‐C was 2–4 times higher than SC at all C rates. The trend was the same for the gravimetric capacities, showing the advantages of DIT‐C at increasing C rates. Upon reverting the C rate back to 0.1 C, the DIT‐C capacity was restored to its initial value, exemplifying the robustness of the DIT method, whereas the inability of the SC electrode to fully recover to its initial capacity upon returning to 0.1 C indicates structural instability of the randomly porous microstructure. Figure S5, Supporting Information summarizes the rate capability of the DIT‐D electrode at increasing C rates, showing that the DIT‐D electrode capacities were consistently higher than SC but lower than DIT‐C. These results demonstrate that the aligned pore arrays in the DIT electrodes improved lithium ion diffusion kinetics through the electrode, particularly at increasing C rates.

Electrochemical impedance spectroscopy (EIS) was applied after three charge and discharge cycles. Figure 7f shows the Nyquist plot of DIT‐C and SC, and the corresponding equivalent circuit is shown in Figure S6, Supporting Information.^[^ ^59^ ^]^ The intercept between the Nyquist plot and Z’ axis at high frequency indicates the resistance of electrolyte *R*_*s*_ and was estimated at 1.9 and 3.5 Ω for the DIT‐C and SC electrodes, respectively. The high‐frequency semicircle and midfrequency semicircle emerged together, where the high‐frequency semicircle diameter indicates charge transfer resistance *R*_CT_ and the midfrequency semicircle diameter can be attributed to the electrolyte‐oxide interfacial impedance *R*_IR_.^[^ ^60^ ^]^ The overall *R*_CT_ and *R*_IR_ were 46.8 and 78.4 Ω for the DIT‐C and SC electrodes, respectively, indicating lower impedance for the aligned microstructure. Figure S7, Supporting Information shows the CV curves of the DIT‐C and SC cathodes at 0.1–0.5 mV s^−1^, where the redox peaks are more prominent in DIT‐C (Figure S7b, Supporting Information) than SC (Figure S7a, Supporting Information). Each peak in the CV curves represents a reduction or oxidation reaction.^[^ ^61^ ^]^ The normalized peak current (*i*_p_) had a linear relation with the square root of the scan rate ($v^{0.5}$) as shown in Figure S8, Supporting Information, indicative of an ion diffusion‐controlled process. The slope values for the cathodic and anodic peaks were similar for the DIT‐C cathode, but asymmetric for the SC cathode, indicative of more reversible redox reactions and faster ion diffusion in both ways in the aligned microstructure of the DIT‐C electrode. The lithium ion diffusion coefficient *D* was calculated by the randles–sevcik equation^[^ ^62^, ^63^ ^]^

(1) $$i_{p}=2.686\times 10^{5}\times n^{\frac{3}{2}}\times A\times D^{\frac{1}{2}}\times C\times v^{\frac{1}{2}}$$

where *i*_p_ is the cathodic or anodic peak current from the CV curve (A), *n* is the number of electrons transferred in the redox event*, A* is the electrode area (cm^2^), *D* is the diffusion coefficient (cm^2^ s^−1^), *C* is the bulk concentration of NMC811 (mol cm^−3^), and *v* is the scan rate (V s^−1^). *D* was 4.2 × 10^−6^ and 2.8 × 10^−6^ cm^2^ s^−1^ for the cathodic and anodic reactions, respectively, for DIT‐C, higher than 1.1 × 10^−6^ and 1.2 × 10^−8^ cm^2 ^s^−1^ for the cathodic and anodic reactions of the SC cathode, respectively. The reduced impedance and enhanced lithium ion diffusion coefficient explained the higher rate capability for the DIT‐C cathode in Figure 7. Finally, Figure S9, Supporting Information shows long cycling performance of the DIT‐C cathode, DIT‐D (with lower mass loading) cathode, and SC (also with lower mass loading) at 0.2 C for 50 times after 70 cycles of rate capability (120 cycles in total). The current of 0.2 C corresponds to discharge current densities of 2.8 and 1.5 mA cm^−2^ for DIT‐C and SC, respectively. The DIT‐C and DIT‐D cathodes exhibited higher capacity retention than the SC cathode, confirming enhanced cycling stability and robustness of DIT method. The DIT‐C and DIT‐D cathodes exhibited similar capacities towards the end, showing water in the low mass loading scenario did not affect NMC811 with any noticeable changes within the rapid DIT processing window before the electrodes were completely dried through ice sublimation. At the high current density, Li metal anode became the limiting factor instead of the cathode. Our future work will optimize the anode.^[^ ^64^ ^]^

To investigate the energy storage performance of the structured electrodes in real‐world devices, we used a Cell Analysis and Modeling System (CAMS) model^[^ ^65^ ^]^ to calculate both gravimetric and volumetric energy density of the batteries using the three types of cathodes in a pouch cell configuration. The schematic diagram of SC and DIT‐C pouch cell configurations (31.9 cm (L) × 23.2 cm (W)) of approximately the same total volume is shown in Figure S10a, Supporting Information. For the SC electrode cell stack in this study (340 μm, deliberately made thicker to compare ion diffusion with the DIT electrodes), 12 layers of cathode and 12 layers of anode were used, whereas an 8‐layer configuration was used for the DIT‐C electrode (660 μm). Figure S10b, Supporting Information shows the volume contributions of each component in SC and DIT‐C‐based pouch cell stacks. In addition, a 5.5 layer configuration was used for the DIT‐D electrode (1074 μm), and a 16.6 layer configuration was used for the conventional SC electrode from the literature (160 μm), to keep the total volume of the pouch cells approximately the same. Table S1, Supporting Information shows the properties of the other components inside the pouch cells, including copper foil with connecting tab, graphite anode, separator, and aluminum foil with connecting tab, while Table S2, Supporting Information shows the proportions of these components inside the cell stacks in a pouch cell for real‐world applications. Figure S10, Supporting Information shows directly that as the DIT electrode thickness increased (with vertically aligned pore arrays to speed up ion diffusion in thick electrodes), the proportion of active materials in cell stacks, and hence, battery energy density, increased. **Table** **1** summarizes the gravimetric and volumetric energy densities of the four types of electrodes (DIT‐C, DIT‐D, SC in this study, and SC in the literature) at different C rates. As shown in Figure S9b, Supporting Information), the DIT‐C cell contained 68.85% active materials by volume, compared to 53.33% in the SC cell. This represents a 15.52% increase in the volumetric allocation for active materials in the DIT‐C cell within the same total volume. Consequently, as shown in Table S2, Supporting Information, the DIT‐C electrode stack contains only 5.07% inactive components, compared to 7.46% in the SC stack. This means the DIT‐C stack reduces inactive component usage by 32% than that in the SC cell stacks, contributing to an overall enhancement in the energy density of the battery cell stack. As a result, DIT‐C exhibited a competitive gravimetric energy density of 533.5 Wh kg^−1^, and a 27% higher volumetric energy density of 1162.2 Wh L^−1^ compared with SC in this study and SC from the literature at 0.1 C. However, as soon as the C rate was increased to 0.4 C, DIT‐C exhibited three times higher gravimetric energy density of 496.2 Wh g^−1^ and over three times higher volumetric energy density of 1080.9 Wh L^−1^ than the SC in this study.

**Table 1:** Summary of gravimetric and volumetric energy densities of the cathodes in battery pouch cell configurations.

C rate [h^−1^]	Energy density	Cathode			
		DIT‐C	DIT‐D	SC (in this study)	SC (from the literature^[^ ^57^ ^]^)
0.1	Gravimetric (Wh kg^−1^)	533.5	321.1	511.1	464.8
	Volumetric (Wh L^−1^)	1162.2	587.1	1076.5	914.1
0.4	Gravimetric (Wh kg^−1^)	496.2	278.2	163.5	418.3
	Volumetric (Wh L^−1^)	1080.9	510.7	344.5	822.7
Aligned electrode microstructure?		√	√	×	×
Electrode thickness [μm]		660	1074	340	160
Mass loading [mg cm^−2^]		70	70	36	17
Average porosity [%]		35	57	35	35
Pore size [μm]		2–6	2–10	2–30	2–30

## 3 Conclusions

A novel DIT method is developed for making anisotropic electrode microstructure with aligned, interleaving lamellae of electrode material and pore arrays to provide fast dual electron and ion transport kinetics. This aqueous DIT method eliminates the toxic, combustible, organic solvent NMP and is more sustainable and safer than the conventional SC method. DIT also enables modification of the fabricated electrode microstructure to improve electrochemical energy storage performance, particularly at faster discharge rates. By controlling the DIT processing, the results of a range of surface‐sensitive techniques (TEM, ToF‐SIMS, XPS) show that the properties of the NMC811 particle surface were similar to that of pristine NMC811 particles in ambient environment and no significant electrochemical performance degradation was observed, demonstrating that water can serve as a viable alternative to NMP in the rapid DIT method. This significantly reduces the environmental impact and health risks associated with the use of NMP, thereby enhancing the sustainability of the battery manufacturing process. We also systematically reduced the porosity of the DIT electrodes to 35%, compatible with the conventional SC electrodes. Due to the almost doubling of the mass loading of the DIT electrodes with the designed microstructure compared with the SC electrode, the DIT‐C electrode exhibited 3 times higher gravimetric capacity and 5 times higher areal capacity than the SC electrode at a discharge current density of 5.7 mA cm^−2^. CV and EIS measurements also show an improved ion diffusion coefficient and reduced impedance for the DIT‐C electrode. Finally, the pouch cell configuration of DIT‐C electrode exhibited superior gravimetric and volumetric energy densities of 496.2 Wh kg^−1^ and 1080.9 Wh L^−1^ at 5.7 mA cm^−2^ compared with 163.5 Wh kg^−1^ and 344.5 Wh L^−1^ for the SC electrodes, with approximately the same total pouch cell volume, because the aligned microstructure improves dual electron and lithium ion transport kinetics during (dis)charging and discharging, while the higher thickness and mass loading reduce the proportion of inactive components (copper foil, aluminum foil, etc.) in the finite volume of pouch cells and increased energy densities.

## 4 Experimental Section

4.1

4.1.1

##### Fabrication of Electrodes

The slurry utilized in the DIT method was prepared under ambient conditions at a room temperature of 15 °C. To ensure the slurry was homogeneous, the suspension was mixed by a planetary mixer (mixer ARE‐250, Thinky company) at a rotation speed of 2,000 rpm for 20 min for both the DIT and SC cathodes. For making cathodes by SC, the process commenced with the mixing of the NMC811 particles, carbon black, and polyvinylidene fluoride binder in the same weight ratio of 90:5:5 in the NMP solvent. The weight ratio between NMP and solid mixture was 50:50.^[^ ^25^ ^]^ Subsequently, the homogeneous slurry was evenly coated onto a flat aluminum foil (16 μm in thickness) at a coating speed of 5 mm s^−1^, employing a doctor blade coater (AFA‐V, Shanghai Modern instruments). Following this, the coated foil was subjected to preliminary drying in an oven at 80 °C for 50 min. The preliminary dried electrode sheet was then transferred to a vacuum oven and exposed to a temperature of 110 °C for 120 min to ensure thorough drying.^[^ ^66^ ^]^ Finally, electrodes with a diameter of 16 mm were precisely cut from the dried electrode sheets using a disc cutter. For DIT, the electrode slurry was rapidly cooled from room temperature down to −40 °C using liquid nitrogen. 30 sets of samples were produced for both DIT and SC cathodes, with the mass loading error controlled within 5%.

##### Characterization

The electrode microstructure was investigated by SEM (Zeiss Sigma300). Cross‐sectional SEM images, were taken after the electrode cross section was polished by a fully automated argon ion milling system (PECS II, GATAN) at 8 keV beam for 5 h to ensure a uniformly flat cross section for subsequent analysis. Segmentation of the cross‐sectional SEM images followed by quantification of the phases, was used to estimate the electrode porosity. TEM (JEOL STEM 2100 Plus), XPS (K‐Alpha), and ToF‐SIMS (ION‐TOF GmbH, Munster, Germany) depth profiling were used to analyze three types of sample: (i) as‐purchased pristine NMC811 particles under ambient conditions without deliberate water exposure (NMC‐ambient conditions); (ii) NMC811 particles that were immersed in water for 30 min (longer than the aqueous slurry mixing duration in the DIT process) (NMC811‐30 min); and (iii) NMC811 particles that were immersed in water for 7 days as a reference for long‐term water exposure (NMC‐7 days). All the samples were then freeze‐dried. To ensure the reliability of these observations, TEM measurements were taken from 10 particles in each sample group, with representative data included in this study. ToF‐SIMS data were acquired using a 25 keV Bismuth liquid metal ion gun analytical beam in positive and negative modes. To obtain high‐resolution mass spectra, a high current bunch mode was used with a beam current of 1 pA. For the ToF‐SIMS depth profile, all samples were sputtered using a 1 keV cesium (90 nA) dual source column ion beam to remove the surface materials layer by layer, and the sputter time had a linear relation with the depth using a constant sputter speed of 0.125 nm s^−1^. The interlaced sputter mode was applied by scanning 100^2^ μm^2^ field at 128^2^ pixels. The XPS measurements (Thermo Scientific K‐alpha) were conducted with 35 eV pass energy for high‐resolution C1s spectra, and Thermo Avantage software was used for data analysis.

##### Electrochemical Testing

To analyze the electrochemical performance of the electrodes, all types of the electrodes made by DIT and SC were transferred into a glovebox (H_2_O level < 0.1 ppm, O_2_ level < 0.1 ppm) and assembled and tested in the standard CR2032 coin cell configuration.^[^ ^57^, ^67^ ^]^ A lithium chip was employed as the counter electrode, lithium hexafluorophosphate solution (1.0 M LiPF_6_ in ethylene carbonate (EC) and ethyl methyl carbonate (EMC) with 50/50 v/v) was used as the electrolyte,^[^ ^8^ ^]^ and a microporous membrane (Celgard 2500) was applied as a separator.^[^ ^68^ ^]^ Galvanostatic charge and discharge were performed from 3.0 to 4.3 V at different C‐rates on a battery cycler (Arbin BT‐G‐48).^[^ ^69^ ^]^ The EIS was investigated from the frequency of 1 MHz to 100 mHz using Biologics SP‐240. CV was performed on Biologic SP‐240 at scan rates of 0.1, 0.2, and 0.5 mV s^−1^. All electrochemical testing was performed at room temperature (15 °C).

## Conflict of Interest

The authors declare no conflict of interest.

## Supporting information

Supplementary Material

## Data Availability

The data that support the findings of this study are available in the supplementary material of this article.
