# Supplementary material for: Battery Cathode with Vertically Aligned Microstructure Fabricated by Directional Ice Templating
Source: Small Sci. 2025 May 22;5(8):2500198. doi: 10.1002/smsc.202500198 (PMC12362797; doi:10.1002/smsc.202500198)
Supplement: Supplementary file 1 — Supplementary Material [file SMSC-5-2500198-s001.pdf]

Supporting Information

Battery cathode with vertically aligned microstructure fabricated by directional ice templating

Guanting Li[a], Jin Su[a,b], Chun Huang[a,b,c*]

[a]*Department of Materials, Imperial College London; London, SW7 2AZ, UK*
[b]*The Faraday Institution; Didcot, OX11 0RA, UK*
[c]*Research Complex at Harwell, Rutherford Appleton Laboratory; Didcot, OX11 0FA, UK*

Corresponding author email address: a.huang@imperial.ac.uk (C. Huang)

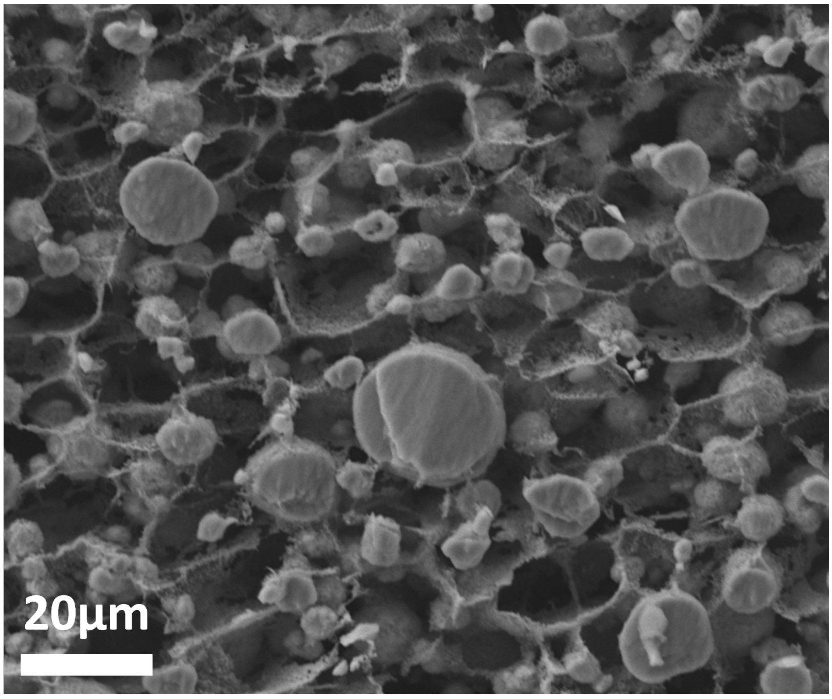

Figure S1. Top view SEM image (after ion milling to reveal the inside microstructure) of DIT-C electrode. The freeze direction was towards out of the page, a cellular structure emerged wherein all active material particles were interconnected in the conductive network of the electrode.

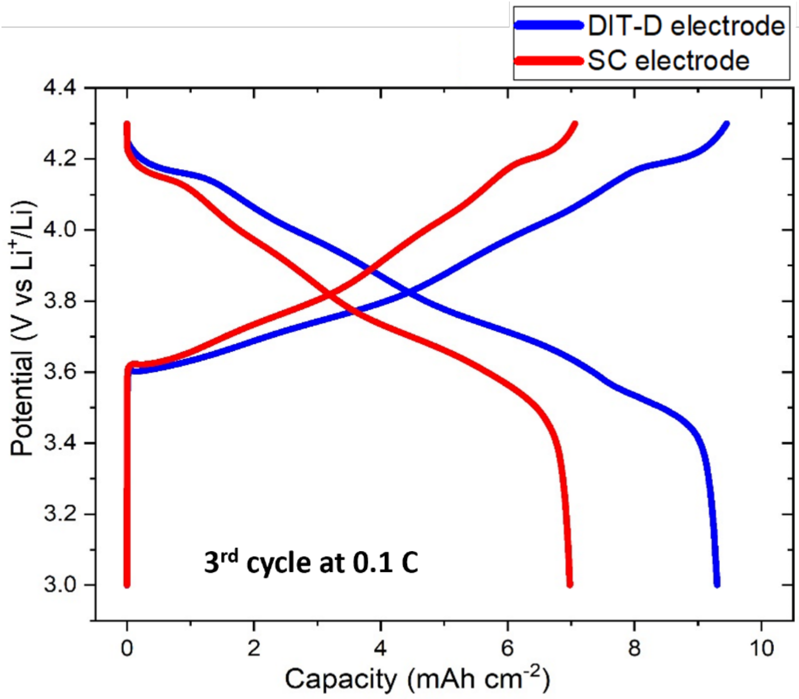

Figure S2. Galvanostatic (dis)charge curves for DIT-D and SC electrodes at 0.1 C.

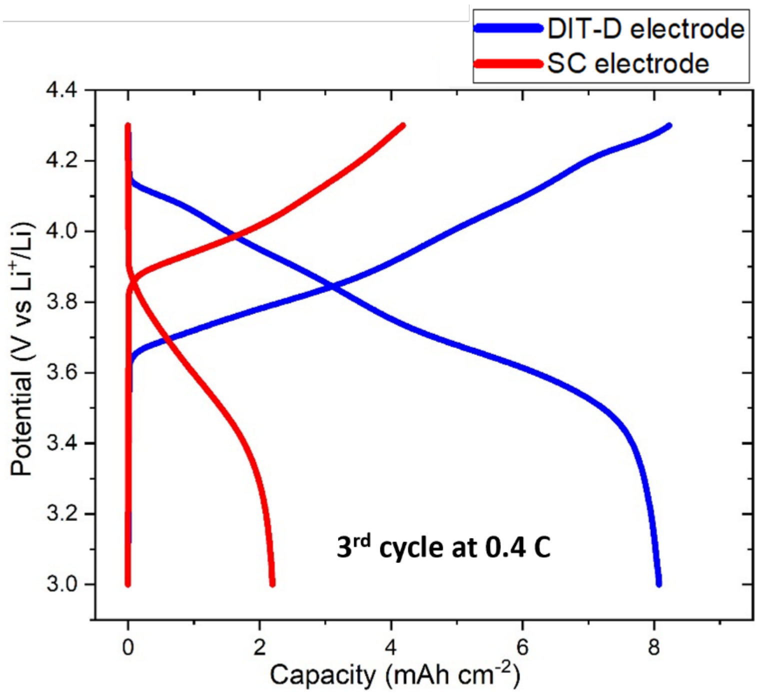

Figure S3. Galvanostatic (dis)charge curves for DIT-D and SC electrodes at 0.4 C.

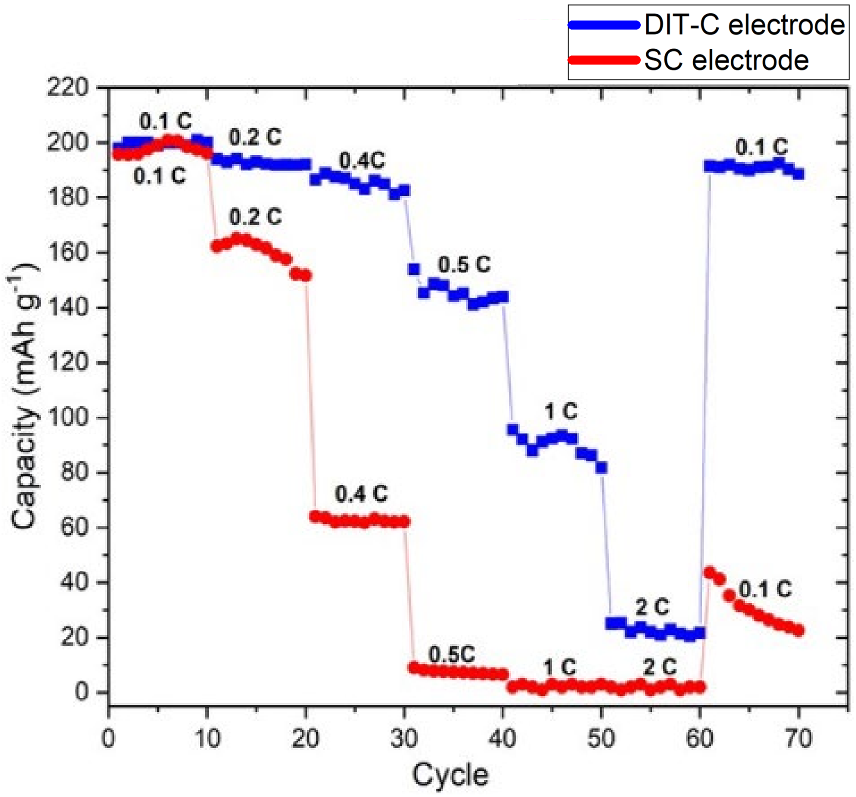

Figure S4. Reversible gravimetric capacity of the DIT-C and SC electrodes from 0.1 C to 2 C.

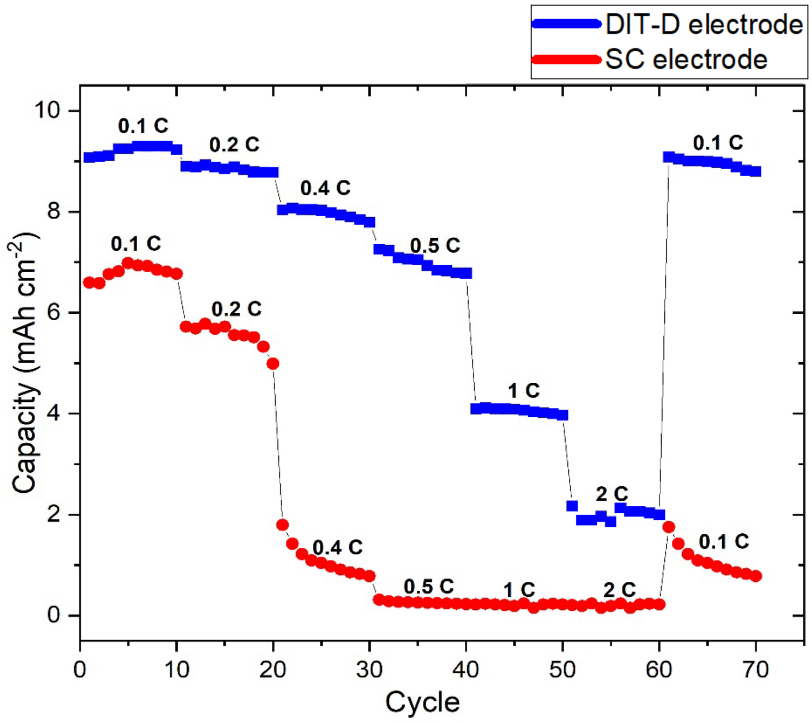

Figure S5. Reversible areal capacity of DIT-D and SC electrodes at different C rates.

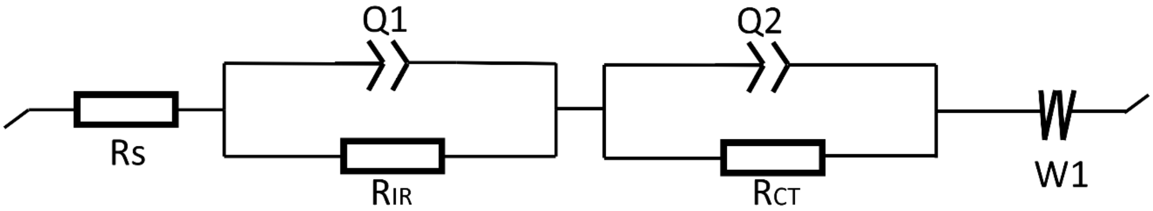

Figure S6. Equivalent circuit for fitting Nyquist Plot of DIT-C and SC electrodes[1].

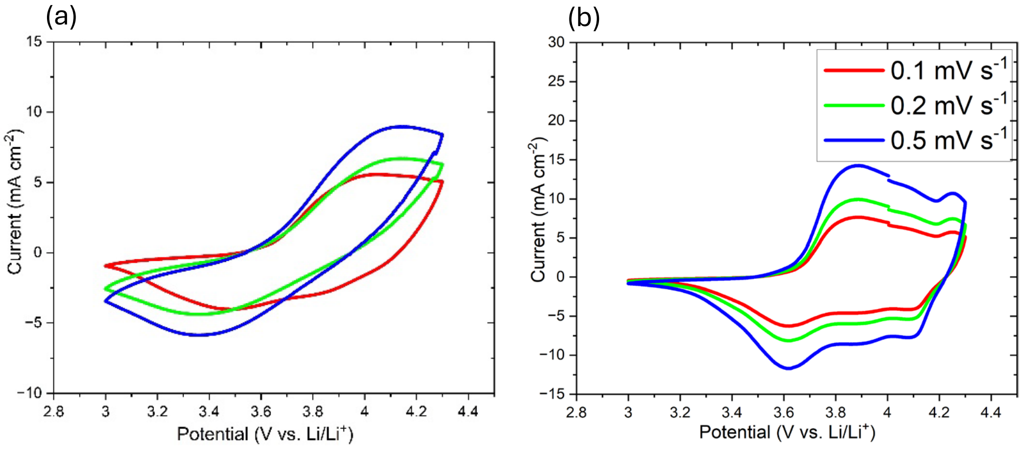

Figure S7. CV curves of (a) SC cathode and (b) DIT-C cathode, at scan rates of 0.1, 0.2, and 0.5 mV s^{-1}.

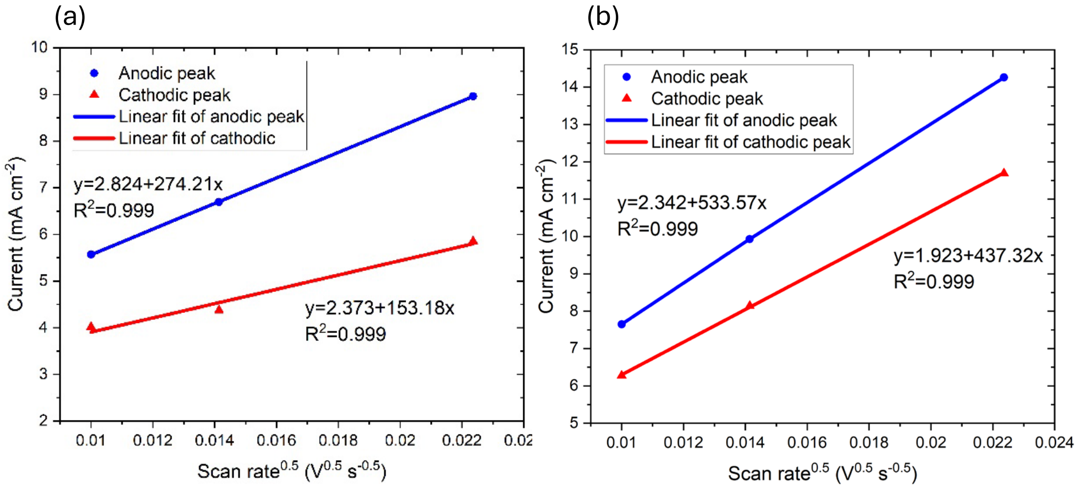

Figure S8. Fitting slop of (a) SC cathode and (b) DIT-C cathode from the normalized peak current of the CV curves.

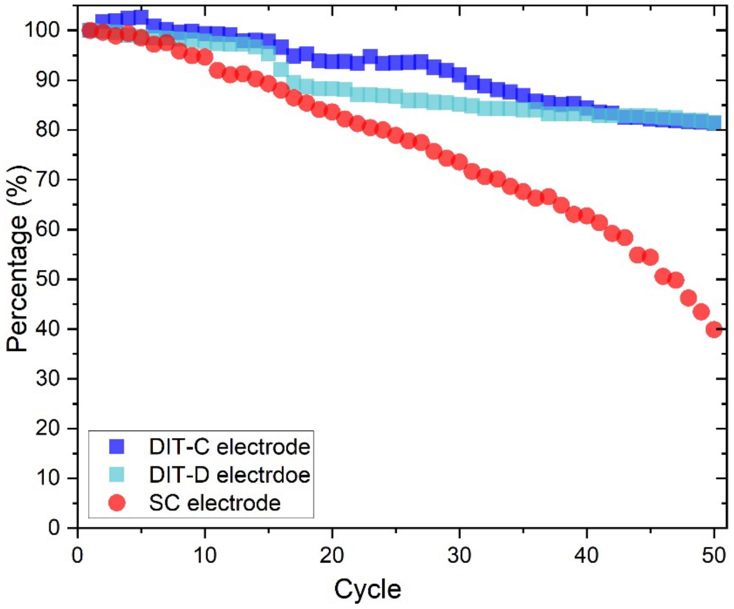

Figure S9. Cycling test of DIT-C, DIT-D and SC electrodes at 2.8 mA cm^{-2} for DIT-C, DIT-D and 1.5 mA cm^{-2} for SC.

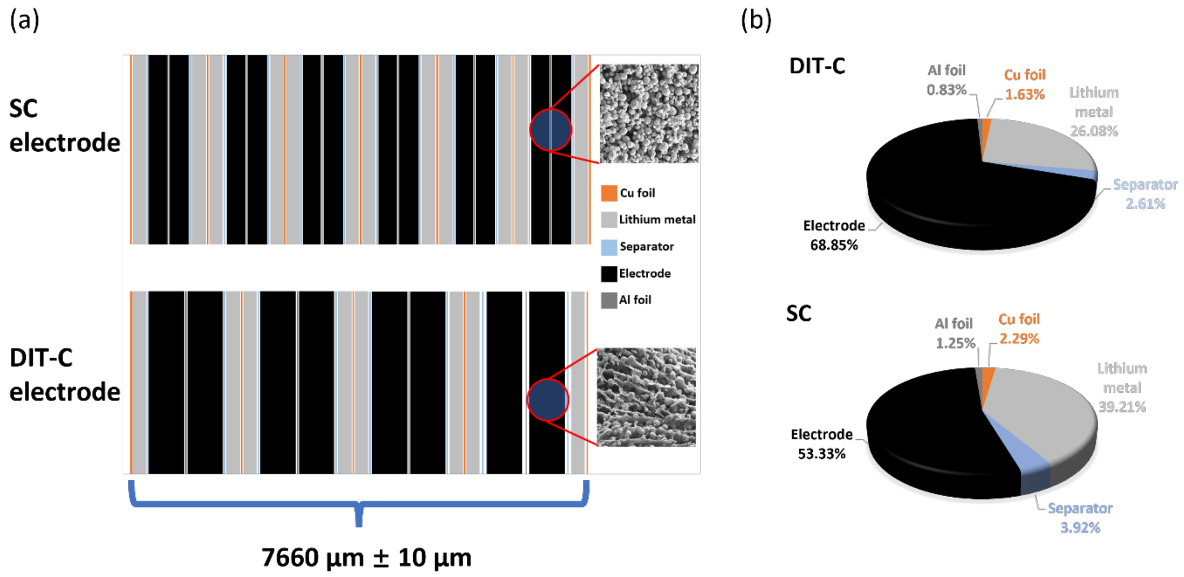

Figure S10. (a)A cross section schematic diagram of SC and DIT-C pouch cell configurations with the same total volume and (b) the volume ratio of components in SC and DIT-C pouch cells.

Table S1. Details of thickness of each component in pouch cell configurations.

Components	Thickness of each component (μm)			Numbers of components			Thickness (μm)		
	12 layers SC electrode	8 layers DIT-C electrode	5.5 layers DIT-D electrode	12 layers SC electrode	8 layers DIT-C electrode	5.5 layers DIT-D electrode	12 layers SC electrode	8 layers DIT-C electrode	5.5 layers DIT-D electrode
Cu foil with tab	25	25	25	7	5	3.75	175	125	93.75
Lithium metal	250	250	250	12	8	5.5	3000	2000	1375
Separator	25	25	25	12	8	5.5	300	200	137.5
Cathode sheet	340	660	1074	12	8	5.5	4080	5280	5907
Al foil with tab	16	16	16	6	4	2.75	96	64	44
				Total thickness (μm)			**7651**	**7669**	**7558**

Table S2. Thickness Distribution of Components in DIT-C vs. SC Cell Stacks

	**SC stack**	**DIT-C stack**	**Difference trend**
Electrode	53.33%	68.85%	↗
Separator	3.92%	2.61%	↘
Lithium metal	39.21%	26.08%	↘
Al foil	1.25%	0.83%	↘
Cu foil	2.29%	1.63%	↘
Inactive component (Separator, Al and Cu foil)	7.46%	5.07%	↘

Reference

1. Vivier, V., Orazem, M.E.: Impedance Analysis of Electrochemical Systems, (2022)
